# A narrative medicine intervention in pediatric residents led to sustained improvements in resident well-being

**DOI:** 10.1080/07853890.2023.2185674

**Published:** 2023-03-04

**Authors:** Nimisha Bajaj, James Phelan, Erin E. McConnell, Suzanne M. Reed

**Affiliations:** a Department of Pediatrics, Nationwide Children’s Hospital, The Ohio State University College of Medicine, Columbus, OH, USA; b Department of English, The Ohio State University, Columbus, OH, USA; c Internal Medicine/Pediatrics, The Ohio State University College of Medicine, Columbus, OH, USA

**Keywords:** Pediatric residency program, narrative medicine, burnout, resident wellness, well-being measures, relationship-centered care

## Abstract

**Background:**

Burnout in pediatric residents is widespread. Certain factors are associated with decreased burnout, such as empathy, self-compassion, mindfulness, and resilience, while perceived stress is associated with increased burnout. Narrative medicine may reduce burnout by its impact on protective and exacerbating factors and can be an active tool to promote wellness. The objective of this pilot study was to evaluate immediate and delayed benefits of a longitudinal narrative medicine intervention for pediatric residents using qualitative and quantitative measures.

**Materials and Methods:**

We designed a voluntary longitudinal narrative medicine intervention implemented *via* Zoom teleconferencing software over five months for pediatric residents at Nationwide Children’s Hospital. It consisted of six one-hour long sessions where residents engaged with literature, responded to a writing prompt, and shared their reflections. It was evaluated using open-ended survey questions and established quantitative assessment tools of well-being with validity evidence. Results were compared before the intervention, immediately after, and six months later using one-way ANOVA and multiple linear regression. Qualitative data was analyzed using thematic analysis.

**Results:**

Twenty-two (14% of eligible) residents participated in at least one session. After the intervention, the following themes emerged for benefits to resident well-being: the ability to *Build Community*, have an *Outlet for Self-Expression*, reap *Emotional and Mental Health Benefits*, and work on *Personal Growth*. Benefits were sustained even six months later, which has not been shown previously. While there were significant qualitative findings, between all three time points, there was no change in any quantitative well-being measures.

**Conclusion:**

Our longitudinal narrative medicine pilot study showed meaningful sustained qualitative benefits, though no quantitative changes, in measured well-being outcomes that have been previously associated with lower resident burnout. While not a panacea, narrative medicine can be a useful strategy for residency programs to improve pediatric resident well-being even after completion of planned interventions.

**Key Message**
- We used a mixed-methods approach to assess the effects of a longitudinal narrative medicine intervention on well-being in pediatric residents.
- Open-ended responses indicated that residents found utility in and appreciated the intervention and experienced sustained improvements in their mental and emotional health, though the sample size was likely too small to show quantitative changes in well-being measures.
- Narrative medicine is not a panacea, but it can be a useful tool to provide to pediatric residents to promote sustained improvements in their well-being through the framework of relationship-centered care.

## Introduction

The incidence of burnout among pediatric residents in the United States is between 43–58% [1–3] and increases over the first six months of intern year, from 17–46% [1]. Many factors have been found to be associated with lower incidence of burnout, including increased empathy, self-compassion, mindfulness, resilience, and confidence in providing compassionate care [2–4]. In one study, perceived stress was the strongest variable associated with risk for burnout [3].

Pediatric residency programs have implemented a variety of interventions to improve trainee wellness, including access to mental health specialists, didactics, debriefing sessions, social events, retreats, and easy availability of exercise equipment [5]. However, many of these interventions do not actively target variables associated with increased burnout.

Narrative medicine is one tool that can be and has been used by individuals and residency programs to target these variables. Narrative medicine is the practice of medicine with narrative competence, or the ability to absorb, interpret, and respond to stories in the context of the relationships between the physician and the patient, the physician and their colleagues, the physician and themselves, and the physician and society [6]. Within the conceptual framework of relationship-centered care, narrative medicine is uniquely suited to build trainees’ ability to positively impact variables that are associated with reduced burnout [7].

Relationship-centered care is based on four interrelated principles that are predicated on the importance of relationships in a healing interaction. The four principles, presented in the context of the physician-patient relationship, are: 1) Relationships include dimensions of personhood, and care interactions require self-awareness because they are relationships between two human beings; 2) Affect and emotion are important components of interactions and relationships in healthcare, so they require emotional presence and empathic response; 3) All health care relationships occur in the context of reciprocal influence, so the practitioner needs to be open to the possibility of growth and transformation; and 4) The cultivation of healing described above is a moral obligation and requires genuine relationships [7,8]. Narrative medicine helps trainees work towards meeting the ideals of relationship-centered care using its own triad of attention, representation, and affiliation [9].

Narrative medicine interventions have been used to promote wellness and empathy and decrease burnout in medical trainees, physicians, and other healthcare providers, though the majority of studies focus on medical students. Generally, programs have a specific format, in which participants read and discuss a short literary piece, do their own short writing in response to a prompt based on the reading, and then share their work, over one or many sessions. For medical students, these interventions have shown qualitative and/or quantitative improvements in empathy, communication skills [10], self-reflection [10–12], active listening and relationship building, mindfulness, observation, perception, and personal growth [12]. However, some studies have had more variable findings. In one study, there was a decrease in self-reported but not quantitatively measured empathy, in participants who did not find the workshop worthwhile, indicating the importance of buy-in [11], and in another there was no significant increase in empathy compared to control [13].

There have been a few published studies on the use of narrative medicine among residents and fellows, ranging from an essay contest for urologists [14] and a single session in a surgical residency [15] to longitudinal interventions for OBGYN residents [16,17], internal medicine residents [18,19], neurology residents [20], and pediatric critical care fellows [21]. These studies generally showed increased empathy and perspective taking [16] and decreased burnout [16,21], as well as improvements in general well-being [18,19] and mindfulness [18]. These interventions also gave participants a sense of joy [15] and meaning in their work [14] and opportunity for self-reflection, listening, and discussion [20].

We designed a mixed-methods pilot study to evaluate the ability of a longitudinal narrative medicine intervention to reduce burnout in pediatric residents through the framework of relationship-centered care. The aim of our study was threefold: (1) to create an intervention that would improve residents’ empathy and mindfulness with downstream effects on improved self-compassion and resilience, and with reduced levels of stress and burnout; (2) to have the beneficial effects of the intervention be sustained beyond the period of the intervention; and (3) to serve as a pilot study to incorporate more robust quantitative analysis of narrative medicine interventions in addition to evaluation of sustained effects and concurrent qualitative analysis.

## Materials and methods

### Overall study design

This was a longitudinal pilot study. Our narrative medicine intervention took place between December 2020 and April 2021. Well-being outcomes were measured before the intervention, immediately after, and six-months later. At the first two time points, we compared quantitative outcomes to those of a control group of residents who did not participate in the intervention. This study was approved by both the Nationwide Children’s Hospital (STUDY 00001270) and Ohio State University (STUDY 2020N0038) Institutional Review Boards.

### Design of intervention

The longitudinal narrative medicine intervention was designed using Kern’s Six Steps [22]. After identifying the overall problem of burnout in pediatric residents as detailed in the introduction (Step 1: Problem Identification and General Needs Assessment), we used a targeted needs assessment (Step 2: Targeted Needs Assessment) to delineate interest in participation, logistics, and self-reported stressors [23]. The objective of the overall intervention was to improve residents’ empathy and mindfulness to eventually lead to reduced stress and burnout (Step 3: Goals and Objectives). NB, JP, and EM devised the sessions using a well-described format for narrative medicine workshops which uses a read-reflect-respond pedagogy (Step 4: Educational Strategies) [12,24]. In each session, participants discussed a short piece of literature, responded to a writing prompt, and shared their writing and reflections with the group. We offered six sessions over five months from December 2020 to April 2021 (two in April), and each one-hour session was offered at both 12:00 pm (during protected time for noon conference) and 6:30 pm. Sessions took place over Zoom teleconferencing software and were led by NB, a peer trained in narrative medicine (Step 5: Implementation). We did not permit pediatrics faculty to attend sessions, though two narrative medicine experts from outside the department, JP and EM, assisted in facilitation of the last two sessions. The literary works in the intervention encompassed themes that related to many stressors identified in the needs assessment. They increased in length over time to reduce initial barrier to participation. A description of the chosen literary works and their associated characteristics is shown in Table S1 (Supplement). We tracked attendance for all sessions. All participants were provided with a notebook for their writing and a copy of *When Breath Becomes Air* by Paul Kalanithi, which was the reading material for the final session.

### Evaluation and feedback (Step 6: evaluation and assessment)

We assessed this intervention *via* participant survey at three time points: T_0_ (pre-intervention), T_1_ (immediately post-intervention completion), and T_2_ (six months post-intervention completion). The survey consisted of the following components: demographic data, existing quantitative well-being assessment tools with validity evidence, and open-ended questions, as described in the following sections.

#### Quantitative survey selection

In order to reduce participant survey burden, we utilized the burnout and resilience survey distributed annually by the Pediatric Resident Burnout-Resilience Study Consortium (PRB-RSC). It has been described in detail previously [2]; it collects demographic data and multiple-choice responses to various well-described well-being measures. In this study, we included demographic data and the following well-being measures: Perceived Stress Scale-10 (PSS-10) [25,26], Cognitive and Affective Mindfulness Scale, R (CAMS-R) [27], Neff’s Self Compassion Scale – Short Form (SCS-SF) [28], Brief Resilience Scale (BRS) [29], Maslach Burnout Inventory – Two Item (MBI) [30], and Davis Empathic Concern Scale from the Interpersonal Reactivity Index (IRI-EC) [31,32]. These six tools were used because we intended to investigate the role of narrative medicine in reducing burnout (MBI). Stress (PSS-10) has been shown to be positively correlated with burnout [3], whereas burnout is negatively correlated with mindfulness (CAMS-R), self-compassion (SCS-SF), resilience (BRS), and empathy (IRI-EC) [2–4]. They are all included into the PRB-RSC study, and all of the residents in our program, in addition to pediatric residents from dozens of programs around the country, are invited to fill out this survey annually. Demographic data was excluded if it would allow for individual identification. We added a multiple-choice question T_0_ to determine past experience in narrative medicine.

#### Qualitative survey design

Open-ended survey questions were only utilized with intervention participants. These questions were designed using best practices [33,34] to assess perceptions of the intervention, including their overall thoughts, perceived benefits, use for their wellness, and suggestions for improvement. We included qualitative questions at T_1_ and T_2_. The full surveys, as they were distributed at each time point, are included in the supplement in Tables S2–S4.

### Participant recruitment and survey administration

All 159 categorical and combined pediatrics (medicine-pediatrics, pediatrics-genetics, pediatrics-neurology) residents at Nationwide Children’s Hospital were eligible to complete the PRB-RSC annual survey. We used convenience sampling because we did not want to impose a wellness intervention, however valuable it has been shown to be, on any of the residents, as the success of a narrative medicine intervention is dependent on participant buy-in and engagement [11]. Participants for the narrative medicine intervention were recruited *via* email over three weeks during December 2020, as well as after each of the first three sessions. We distributed surveys as described in Table 1 to be completed over three to five weeks. The PRB-RSC survey is described above, and the supplement survey contained the qualitative and narrative medicine questions. For the intervention group, if participants had completed the previous year’s burnout and resilience survey, they did not repeat it.

**Table 1.** Survey distribution time points.

Group	T_0_ Pre-Intervention (April–May 2020 or December 2020)	Intervention (December 2020–April 2021)	T_1_ Immediate Post-Intervention (April–May 2021)	T_2_ Six Months Post-Intervention (October–November 2021)
Intervention	PRB-RSC Survey Supplement Survey T_0_		PRB-RSC Survey Supplement Survey T_1_	PRB-RSC Survey Supplement Survey T_2_
Control	PRB-RSC Survey		PRB-RSC Survey	No data

PRB-RSC: Pediatric Resident Burnout-Resilience Study Consortium. Supplement Survey included narrative medicine-specific and open-ended questions.

### Data collection

We collected data at three time points using two systems. The PRB-RSC survey used the Association of Pediatric Program Directors (APPD) Longitudinal Educational Assessment Research Network (LEARN) ID system. The remaining study data were collected and managed using REDCap electronic data capture tools hosted at the Ohio State University [35,36]. REDCap (Research Electronic Data Capture) is a secure, web-based software platform designed to support data capture for research studies, providing 1) an intuitive interface for validated data capture; 2) audit trails for tracking data manipulation and export procedures; 3) automated export procedures for seamless data downloads to common statistical packages; and 4) procedures for data integration and interoperability with external sources.

### Data processing

Quantitative data was either received from the PRB-RSC (if it was collected as part of the annual burnout resilience survey) or downloaded from REDCap (the supplement data or the off-cycle initial post-intervention survey) into Microsoft Excel. Only NB and SR have access to the data in password protected files, which will be kept until publication of the study. An anonymous ID linked scores across time points. This ID was randomly generated for each participant and a table linking the ID to the participant was only accessible to the residency program coordinator, CZ, who did not have access to any of the data and disposed of the table after the last survey data was collected. Demographic data was removed or aggregated as necessary to prevent identifiability. Raw scores of well-being survey measures were processed as appropriate. The PSS-10 [25,26], CAMS-R [27], MBI [30], and IRI-EC^31^ [32], scores were calculated as a sum total while the SCS-SF^28^ and BRS^29^ scores were calculated as item means.

### Statistical analysis

All statistical analysis was done using GraphPad Prism. Attendance was described using descriptive statistics. At T_0_, we compared demographic data between intervention and control groups using a student’s two-tailed unpaired t-test, Fisher’s exact test, or chi-square as appropriate. In the same way, we compared demographic data between those in the intervention group who took the well-being survey at T_0_ in April versus December 2020. Well-being measures were compared at T_0_ using a student’s two-tailed unpaired t-test between control and intervention groups as well as between those in the intervention group who took the initial survey in April versus December, based on demographic variables, and based on previous experience with narrative medicine. In the case of hours worked per week, we used a linear regression to compare well-being measures.

A mixed-effects ANOVA was used to compare well-being measures at T_0_, T_1_, and T_2_ for all participants in the intervention group using a one-way ANOVA. For well-being measures in which there was a statistically significant difference between demographic groups at T_0_, a multiple linear regression was done to compare well-being measures at T_1_ based on dose of intervention (number of sessions attended), controlling for respective demographic variables. Significance in all analyses was defined as *p* < 0.05.

### Thematic analysis

We analyzed open-ended questions from T_1_ and T_2_ using inductive thematic analysis based on a well-accepted framework [37]. Briefly, the data was downloaded from REDCap into Microsoft Excel, with each answer to each question by an individual participant in a cell. We familiarized ourselves with the data. Answers for all the questions were coded together for each participant, so there was only one overall code list. Two investigators (NM, SR) independently coded data and generated their own codes. The code lists were reconciled and the data were recoded iteratively until agreement was reached, yielding a final code list that was used for data for all questions at both T_1_ and T_2_. Codes were then aggregated into subthemes and themes for the two separate groups, which were compared to original data sets to confirm adequate description of the raw data. Each code only needed to be mentioned once in order to be included when determining themes and subthemes.

## Results

### Total study recruitment and session attendance

Out of 159 total residents in the program, 23 (14%) consented to participation in the intervention, and 22 completed the initial survey and attended at least one session. A mean of 10 residents participated in each session (minimum 7, maximum 18), and each resident participated in a mean of 2.56 out of 6 sessions (minimum 0, maximum 6). For the control group, 85 residents (53%) completed the PRB-RSC survey, but did not participate in the intervention, at T_0_, and 79 residents (50%) at T_1_. Eleven of the intervention residents completed the PRB-RSC survey at T_0_ in April 2020, and the remaining 11 in December 2020.

### Baseline demographics

Baseline demographic characteristics for participants in the control and intervention groups are shown in Table 2. The only statistically significant difference in groups was residents’ last rotation, in which more control participants were on a primary care or elective rotation and more intervention group participants were on an inpatient, newborn nursery, or night float rotation (*p* = 0.01). Additionally, average hours worked per week in the month prior to completing the survey was 60.4 h (SD 9.07 h) for the intervention group and 52.3 h (SD 11.1 h) in the control group with *p* = 0.002. There was no difference in baseline demographics between intervention group participants who took the initial survey in April versus December 2020 (Table S5).

**Table 2.** Baseline demographic data of participants in the control and intervention groups.

Category	Group	Intervention *n* (%)	Control *n* (%)	*p* Value
Gender	Male	5 (24)	25 (31)	0.60
	Female	16 (76)	56 (69)	
	Total	21	81	
Age	<30	18 (86)	62 (79)	0.76
	30+	3 (14)	16 (21)	
	Total	21	78	
International Medical Education	Yes	1 (5)	0 (0)	0.22
	No	21 (95)	78 (100)	
	Total	22	78	
Marital Status	Single/divorced	10 (45)	44 (52)	0.64
	Married/partnered	12 (55)	41 (48)	
	Total	22	85	
Children	Yes	2 (10)	6 (7)	0.67
	No	19 (90)	75 (93)	
	Total	21	81	
Categorical	Yes	16 (73)	69 (81)	0.39
	No	6 (27)	16 (19)	
	Total	22	85	
Last Weekend Off	Previous weekend	9 (41)	47 (55)	0.44
	2 weekends ago	8 (36)	21 (25)	
	3+ weekends ago	5 (23)	17 (20)	
	Total	22	85	
Last Vacation	In the past month	9 (41)	25 (29)	0.47
	1–3 months ago	6 (27)	34 (40)	
	>3 months ago	7 (32)	26 (31)	
	Total	22	85	
Last Rotation	Inpatient/Newborn/Nightshift	12 (57)	19 (24)	***0.01***
	ER/ICU	4 (19)	17 (22)	
	Primary Care/Elective	5 (24)	43 (54)	
	Total	21	79	
Patient Death	Yes	2 (9)	18 (21)	0.24
	No	20 (91)	67 (79)	
	Total	22	85	

*Note: n* may not sum to total sample size because of missing data. Percentages may not sum to 100% due to rounding. p-values are bolded and italicized if p<0.05.

### Comparison of well-being measures across demographics

Baseline values of all well-being measures compared across all demographic variables are shown in Table 3. Status as an international medical graduate was not included because there was only one participant in that group. For hours worked per week in the last month, there was no statistically significant difference in any well-being measures.

**Table 3.** Baseline well-being measures compared across demographic groups and previous experience with narrative medicine.

			Well-Being Measure Average (Standard Deviation)					
Variable	Group	*n*	PSS-10	CAMS-R	SCS-SF	BRS	IRI-EC	MBI
Intervention vs. Control	Intervention	22	17.0 (5.6)	28.0 (3.5)	3.2 (0.53)	3.6 (0.65)	24.7 (3.1)	5.0 (3.0)
	Control	85	14.4 (5.9)	28.4 (4.7)	3.3 (0.58)	3.7 (0.59)	22.9 (3.7)	3.9 (2.5)
	*p*-value		0.08	0.73	0.34	0.54	***0.04***	0.10
Intervention April vs. December Survey	April	11	17.5 (4.0)	28.1 (2.9)	3.3 (0.30)	3.7 (0.47)	24.4 (3.3)	5.5 (2.7)
	December	11	16.5 (6.9)	27.9 (4.1)	3.0 (0.67)	3.5 (0.81)	25.1 (3.0)	4.5 (3.2)
	*p*-value		0.68	0.91	0.27	0.63	0.60	0.44
Gender	Male	30	13.8 (6.6)	28.6 (4.8)	3.4 (0.54)	3.8 (0.64)	22.0 (3.2)	4.0 (2.6)
	Female	72	15.5 (5.7)	28.4 (4.1)	3.2 (0.57)	3.6 (0.57)	23.9 (3.8)	4.3 (2.7)
	*p*-value		0.19	0.78	0.24	0.20	***0.02***	0.61
Age	<30 years	80	15.6 (5.8)	27.7 (4.2)	3.2 (0.54)	3.7 (0.58)	23.0 (3.9)	4.5 (2.8)
	30+ years	19	13.3 (5.5)	29.2 (5.0)	3.3 (0.65)	3.6 (0.75)	24.0 (2.8)	3.3 (1.8)
	*p*-value		0.14	0.17	0.41	0.50	0.29	0.07
Marital Status	Single/Divorced	54	15.1 (5.2)	28.0 (3.8)	3.2 (0.53)	3.6 (0.57)	23.4 (3.6)	4.2 (2.6)
	Partnered/Married	53	14.8 (6.6)	28.6 (5.0)	3.3 (0.61)	3.8 (0.62)	23.2 (3.7)	4.1 (2.7)
	*p*-value		0.81	0.42	0.47	0.20	0.78	0.86
Children	Yes	8	11.0 (3.4)	30.3 (1.8)	3.4 (0.43)	4.0 (0.25)	23.1 (3.0)	3.8 (1.3)
	No	94	15.5 (5.9)	27.8 (4.3)	3.2 (0.56)	3.6 (0.60)	23.2 (3.8)	4.3 (2.7)
	*p*-value		***0.048***	0.11	0.30	0.08	0.96	0.60
Categorical Resident	Yes	85	15.4 (6.1)	27.9 (4.6)	3.2 (0.61)	3.7 (0.64)	23.4 (3.6)	4.1 (2.8)
	No	22	13.2 (4.8)	29.8 (3.3)	3.4 (0.39)	3.7 (0.44)	23.0 (4.0)	4.1 (2.1)
	*p*-value		0.13	0.08	0.38	0.85	0.61	0.95
Last Weekend Off	Previous Weekend	56	14.6 (6.1)	27.9 (4.6)	3.2 (0.62)	3.6 (0.61)	23.0 (4.0)	3.9 (2.6)
	2 Weekends Ago	29	15.7 (5.6)	28.3 (4.2)	3.2 (0.48)	3.8 (0.49)	23.9 (3.1)	4.4 (2.9)
	3+ Weekends Ago	22	15.0 (6.1)	29.3 (4.3)	3.4 (0.53)	3.7 (0.70)	23.3 (3.3)	4.4 (2.4)
	*p*-value		0.73	0.46	0.29	0.49	0.54	0.56
Last Vacation	In the past month	34	15.3 (6.2)	27.8 (4.8)	3.2 (0.58)	3.6 (0.62)	23.5 (3.3)	4.2 (2.7)
	1–3 months ago	40	14.6 (5.1)	28.9 (4.2)	3.3 (0.50)	3.7 (0.57)	23.6 (3.7)	4.2 (2.4)
	>3 months ago	33	15.0 (6.7)	28.1 (4.3)	3.3 (0.64)	3.7 (0.62)	22.8 (4.0)	3.9 (3.0)
	*p*-value		0.90	0.57	0.48	0.80	0.65	0.86
Last Rotation	Inpatient/ Newborn/Night Shift	31	14.9 (6.5)	28.5 (4.7)	3.2 (0.67)	3.6 (0.65)	24.3 (3.3)	4.0 (2.8)
	ER/ICU	21	16.9 (7.0)	29.0 (4.6)	3.3 (0.60)	3.8 (0.72)	24.4 (3.2)	4.3 (2.6)
	Primary Care/Elective	48	14.2 (4.8)	28.0 (4.4)	3.3 (0.50)	3.7 (0.53)	22.3 (3.8)	4.0 (2.6)
	*p*-value		0.25	0.72	0.68	0.71	***0.02***	0.89
Patient Death in the Last Month	Yes	20	14.0 (5.0)	29.1 (4.1)	3.4 (0.44)	3.8 (0.62)	22.4 (4.0)	4.6 (2.2)
	No	87	15.2 (6.1)	28.1 (4.5)	3.2 (0.59)	3.7 (0.59)	23.5 (3.6)	4.0 (2.7)
	*p*-value		0.43	0.40	0.26	0.44	0.22	0.37
Previous Experience with Narrative Medicine	None	14	17.6 (6.1)	27.4 (3.2)	3.1 (0.45)	3.7 (0.69)	23.8 (3.1)	5.2 (2.6)
	I have participated in a few workshops	9	16.1 (5.0)	28.9 (3.9)	3.2 (0.65)	3.5 (0.60)	26.1 (2.7)	4.6 (3.6)
	*p*-value		0.57	0.33	0.67	0.58	0.08	0.61

PSS-10: Perceived Stress Scale-10; CAMS-R: Cognitive and Affective Mindfulness Scale, R; SCS-SF: Neff’s Self Compassion Scale – Short Form; BRS: Brief Resilience Scale; IRI-EC: Davis Empathic Concern Scale from the Interpersonal Reactivity Index; MBI: Maslach Burnout Inventory – Two Item. Note: n may not sum to total sample size because of missing data. p-values are bolded and italicized if p<0.05.

Only IRI-EC and PSS-10 varied based on any demographic variables in control versus intervention group, and there was no difference in baseline well-being measures between participants in the intervention group who took the baseline survey in April versus December 2020. Lower baseline scores for empathic concern were found in participants in the control group (*p* = 0.04), males (*p* = 0.02), and participants who were on a primary care rotation or elective (*p* = 0.02). Baseline perceived stress scale scores were lower in those who reported having children (*p* = 0.048), though the sample size for parents was *n* = 8 and for non-parents was *n* = 94.

### Previous experience with narrative medicine

Among the 23 participants of the intervention, 14 had no previous experience and 9 had some experience with narrative medicine. No one practiced narrative medicine regularly. There was also no statistically significant difference in well-being measures based on previous experience with narrative medicine (Table 3).

### Quantitative change in well-being measures

Sixteen residents completed the survey without missing data points at all three time points so were included in the analysis. A one-way ANOVA comparing values of each of the well-being measures across time for intervention participants showed no statistically significant difference between time points for any of the scales (Table 4).

**Table 4.** Results of one-way ANOVA comparing well-being measures in intervention group at each time point.

	Average Value (Standard Deviation) at Each Time Point			
Well-Being Measure	T_0_	T_1_	T_2_	*p* Value for Fixed Effects
PSS-10	17.0 (5.6)	16.1 (4.7)	15.6 (5.9)	0.26
CAMS-R	28.0 (3.5)	28.7 (5.0)	27.7 (5.3)	0.64
SCS-SF	3.2 (0.53)	3.2 (0.49)	3.2 (0.52)	0.71
BRS	3.6 (0.65)	3.7 (0.77)	3.6 (0.63)	0.70
IRI-EC	24.7 (3.1)	25.4 (2.9)	24.7 (3.5)	0.58
MBI	5.0 (3.0)	4.6 (2.7)	5.3 (3.3)	0.34

PSS-10: Perceived Stress Scale-10; CAMS-R: Cognitive and Affective Mindfulness Scale, R; SCS-SF: Neff’s Self Compassion Scale – Short Form; BRS: Brief Resilience Scale; IRI-EC: Davis Empathic Concern Scale from the Interpersonal Reactivity Index; MBI: Maslach Burnout Inventory – Two Item.

A multiple linear regression was utilized for the two outcome measures, IRI-EC and PSS-10, that were susceptible to confounding by demographic variables. This analysis included both control and intervention data only at T_1_, where number of doses was set to zero for participants in the control group. There was no significant difference in perceived stress or in empathic concern in participants at T_1_ who received different doses of the intervention when controlling for having children or not (PSS-10) or gender and their last rotation (IRI-EC).

### Resident perceptions of Intervention

Results of the thematic analyses of perceptions of the intervention at T_1_ and T_2_ are shown in Table 5. The main theme regarding overall perception was that the intervention was *Enjoyable and Well-Planned*. For present and future benefits, the predominant themes were the ability to *Build Community*, have an *Outlet for Self-Expression*, reap *Emotional and Mental Health Benefits*, and work on *Personal Growth*. Themes and subthemes regarding overall perception of the intervention and present and future benefits were all elicited at both T_1_ and T_2_. The theme of *Delayed and Persistent* Benefits was only identified at T_2_. The two main themes associated with areas of improvement, only assessed at T_1_, were *Program Expansion and Program Adjustment to Learner Needs*.

**Table 5.** Resident perceptions of intervention at T_1_ and T_2_.

Theme	Subtheme	Representative Quote(s)
**Overall Thoughts about the Intervention (Identified at T_1_ and T_2_)**		
Enjoyable and Well-Planned		‘I really enjoyed participating in the Narrative Medicine sessions, and I think this type of intervention could be helpful for all residents. I really appreciate that we could take what we needed from the different exercises, and this flexibility is so important in promoting overall wellness.’
**Present and Future Benefits of the Intervention (Identified at T_1_ and T_2_)**		
Building Community	Safe Space	‘I feel like it helped me realize that I can be honest about my feelings and thoughts with those around me.’
	Shared Experiences	‘I feel reassured that I am not alone in my experiences.’
Outlet for Self-Expression		‘Enjoyed thinking creatively. Just writing for fun without any expectations was freeing.’
Emotional and Mental Health Benefits	Reduced Stress	‘I felt more relaxed and at ease after the sessions, and I think this was because I was able to spend time expressing feelings and experiences with peers who often times have gone through similar things.’
	Reflection	‘I feel like it made me think more about the thoughts/feelings that I suppress as we do not have a structured outlet to really understand what all is going on in our minds.’
	Emotional Regulation	‘I feel like talking through tough topics is helpful for decompressing and organizing my feelings. It overall helped me to try to be more thoughtful with all my hard/emotional patient encounters.’
Personal Growth		‘I genuinely loved these sessions. I found them to be strength building for me as a human, and especially so in coping with residency.’
Delayed and Persistent Benefit (Identified at T_2_)		‘I think it had a lasting impression.’ ‘I thought it helped me later than actually earlier. These past 6 months have been harder and reading and letting myself have my own feelings has really helped me.’
**Areas of Improvement (Identified at T_1_)**		
Program Expansion	Multiple Settings	‘I would love for these sessions to be included in retreats where you’re among residents in the same year as you.’ ‘Maybe find ways to incorporate this into small sessions while on inpatient?’
	Program Continuation	‘If the program could be expanded so more residents could participate that would be great.’ ‘I would love to see this continued.’
	Protected Time	‘I wish there was protected time for this program. Many of the times I was on service and even if it was at our ‘protected’ 12-1, I was still interrupted and the evening sessions were also interrupted by night shift/other obligations.’
Program Adjustment to Learner Needs	More Intimacy	‘Groups can be made smaller, the session I attended had a lot of people, and I didn’t really feel that comfortable sharing.’ ‘I wish we could have been in person!’
	Increase Accessibility	‘It could be improved by allowing by additional shorter articles to be discussed for those on inpatient medicine and have more small group opportunities.’ ‘Trying to have a curriculum that’s more learner-paced?’

## Discussion

This pilot study aimed to assess the immediate and delayed effects of a longitudinal narrative medicine intervention on well-being in pediatric residents, measured both quantitatively using well-being survey measures with validity evidence and qualitatively using thematic analysis of open-ended responses. We anticipated that narrative medicine could have effects on well-being through the framework of relationship-centered care, as the engagement with literature and reflection in small group settings would facilitate relationship-building for residents. We found that there were no statistically significant changes in any of the quantitative well-being measures over time, even when correcting for possible confounding from demographic and lifestyle variables. However, through the thematic analysis, the data showed meaningful and persistent benefits for residents in the ability to *Build Community,* have an *Outlet for Self-Expression*, have *Emotional and Mental Health Benefits*, and work on *Personal Growth*.

### Quantitative well-being measures

In this study, we did not find any significant changes in any of the quantitative well-being indices over the course of the intervention or afterwards. The lack of statistically significant changes could be due to a number of factors. With data from only 16 residents analyzed, the study was likely underpowered. Additionally, when we controlled for demographics in a multiple linear regression, we still found no changes in well-being measures over the course of the intervention or afterwards, and demographic variables had no significant effects on the models. Especially in the middle of a pandemic, much of this lack of quantitative difference could be due to variables that we did not account for affecting well-being measures. And while there was no difference in baseline characteristics between participants of the intervention who took the T_0_ survey in April versus December, there could have been changes for individual participants during that time.

Other interventions using narrative medicine in residents have found quantitative increases in empathic concern and perspective taking and a decrease in emotional exhaustion for high attendance participants [16], as well as less of a decrease, though not significantly, in well-being over the course of a difficult rotation compared to control [19]. In medical students, they have shown significant increases in empathy and reflection scores [10]. However, another study also showed no significant changes over time in empathy [13], like we did, and we incorporated significantly more confounders, including age, parental status, gender, and marital status, than any of these studies, though we were limited by a small sample size.

### Qualitative reporting of benefits

The qualitative results, importantly, indicate the intervention had positive effects. Relationship-centered care is based on the idea that building genuine relationships is a moral obligation for physicians, and it requires building empathy, openness to personal growth, and treatment of the physician and patient as whole people. These are all integral to well-being, and our study indicated that narrative medicine could be used achieve these goals.

As determined by the open-ended survey questions, residents self-reported sustained benefits in reduced stress, mindfulness, decreased burnout in the form of improved emotional and mental health, improved empathy, self-compassion through reflection, community building, perspective-taking, and resilience through having narrative medicine as a new coping skill that they could take with them and use in the future. The aforementioned themes tie to the four principles of relationship-centered care [7]. Reflection and perspective-taking increase self-awareness as well as emotional presence and empathy. Our participants expressed appreciation for developing a new skill, which shows their commitment to serve and grow and their openness to transformation, along with improved mindfulness.

Other studies in residents and medical students have found similar themes, including creating a safe space [17], and increasing mindfulness [12,18], reflection [12,18,20], empathy, emotional processing and well-being [18], and a sense of community through building relationships [12,18]. These interventions have mostly been well-received and have shown to increase joy and meaning in work and ability to serve patients [12,15,20]. In one study, similar to our own results, many participants also commented that narrative medicine facilitated significant personal growth [12]. Notably, these studies only assessed immediate benefits of the intervention, as opposed to prolonged utility for participants.

### Significant contributions

The most significant contribution of our study is that we reported delayed and persistent benefits of a narrative medicine intervention, which is a unique addition to the literature. No other studies have measured sustained benefits after the conclusion of the intervention. This result highlights not only that narrative medicine is something that benefits residents in the moment, but that it is a tool that they can carry with them months into the future to support their well-being. Residents’ schedules are incredibly busy, so they do not always have the availability to participate in organized programs, and these sustained improvements in coping and self-reflection can be advantageous. Furthermore, many comments regarding areas of improvement indicated an interest in continuing and expanding the program, which may lead to even more prolonged benefits. Another strength of our study is the fact that we furthered the use of quantitative well-being measures in this type of work.

### Future work

Future work in this area should have three main components. First, it should optimize the intervention itself to meet learner needs, having smaller groups, assuring protected time, and adapting it to the level and location of the participant, in order to maximize the possible impact. It should also, as we did, choose well-crafted narratives for the intervention that are relevant to the experiences of the participants. Second, it should expand the reach of the intervention over multiple years, to other groups such as fellows, or to other residency programs either nationally or within the institution, which would be possible with the use of teleconferencing software. Thirdly, it should continue to assess quantitative well-being measures and evaluate sustained benefit, but with a larger sample size and with an effort to isolate the effects of more confounding variables which would allow for deeper analysis of the well-being measures.

## Limitations

This pilot study had the following limitations:

- The major limitations of our study were the small sample size and the limited number of sessions attended by participants. As mentioned previously, our sample size of 23 residents (16 in the final analysis) was similar to other interventions using narrative medicine at individual residency programs [18–20]. However, with the quantitative data, the small sample size of the intervention group compared to control may have skewed the statistical analysis.
- Participation was voluntary, which limited sample size and generalizability of results to those who are less familiar with narrative medicine, but we did not want to force participation in a well-being intervention, since self-care is so personal. While narrative medicine is also used for professional development, which is inextricably linked with personal well-being, we centered our investigation and focus on reducing burnout. Ergo, we were able to limit involvement to those who were truly invested in the intervention. Additionally, conducting the study within a close-knit residency class allowed us to create a safe, more comfortable space that facilitated robust discussion.
- Participants also mentioned that they wish the study had been done in person, though we needed to conduct the intervention over Zoom teleconferencing software due to the constraints of the COVID-19 pandemic. While we indicated during the consent process that every participant should join on their own computer in a private area where they could concentrate, that may not have been possible for every participant. While it increased accessibility for residents to join remotely if they were offsite or at home, it may have affected their ability to fully participate if they were joining from a hospital unit or had children or other family members in the background. Other studies of virtual education in residents have shown that there are limitations to not being in a single location, especially as the time is sometimes not protected [38], and it can affect learning, but while they prefer in-person education, overall virtual education experiences can be nearly as effective [38,39].
- For the sake of reducing survey burden, we also limited the number of demographic and confounding variables assessed to those already present on the PRB-RSC survey, which may have hampered our ability to assess changes in well-being measures. While we did include previous training or utilization of narrative medicine, we did not account for other mindfulness or relaxation techniques or generally what else residents did for their own wellness.
- Additionally, per participant comments, they mentioned a desire for smaller groups and having protected time and shorter articles, which could have suggested they were limited in participation by the internal setting of the intervention or their external environment.

## Conclusion

In this study of a longitudinal narrative medicine intervention for pediatric residents at Nationwide Children’s Hospital, we found that despite no significant changes in quantitative measures of perceived stress, self-compassion, empathy, mindfulness, burnout, or resilience over time, residents showed sustained self-reported qualitative improvements on those same measures that lasted over at least six months. Many of these well-being measures have been associated with reduced burnout, but also through the framework of relationship-centered care, with improved physician relationships and patient care, indicating that narrative medicine is a tool that other residency programs can use as well. Most importantly, this is the first time a study has shown persistent improvement of well-being in residents using narrative medicine, and it indicates that residents do not have to commit to the time of a structured intervention year-round to reap the benefits.

## Supplementary Material

Supplemental Material

## Data Availability

The data that support the findings of this study are openly available in Open ICPSR at https://doi.org/10.3886/E170961V1, reference number 40 [40].
