# Supplementary material for: A narrative medicine intervention in pediatric residents led to sustained improvements in resident well-being
Source: Ann Med. 2023 Mar 4;55(1):849–59. doi: 10.1080/07853890.2023.2185674 (PMC9987757; doi:10.1080/07853890.2023.2185674)
Supplement: Supplemental Material [file IANN_A_2185674_SM2733.pdf]

Supplemental Tables

Table S1: Intervention Content

Ses.	Literary Work	Genre	Length of Work	Stressors	Writing Prompt
1	"How is Residency?" by Mike Natter, MD[1]	Graphic Medicine	Single Frame	• Lack of or Poor Sleep<br>• Staying Physically Healthy<br>• Finances<br>• Loneliness and Social Isolation<br>• Work-Life Balance	"Talk about a time when you felt left behind."
2	"Migraine" by Jessica Hernandez[2]	Graphic Medicine	Multiple Frames	• Staying Physically Healthy<br>• Loneliness and Social Isolation<br>• High Work Load and Expectations<br>• Negative Work Environment	"Write about a time when you did or wish you had made some noise."
3	"Keep That Same Energy" by Alexandra Sims, MD[3]	Poem	1 Page	• Moral Distress and Empathy Fatigue	"Write about your silent prayers for your patients."
4	"The Steel Windpipe" from *A Country Doctor's Notebook* by Mikhail Bulgakov	Short Story	8 Pages	• Lack of or Poor Sleep<br>• Loneliness and Social Isolation<br>• Sick/Dying and Difficult Patients<br>• Work-Life Balance<br>• Unusual, Variable, and Uncertain Work Schedule<br>• High Work Load and Expectations<br>• Lack of Confidence and Feelings of Inadequacy	"Write about the first time you did something important and intimidating."
5	*Wit* directed by Mike Nichols	Feature Film	1 Hour, 39 Minute Film	• Moral Distress and Empathy Fatigue<br>• Sick/Dying and Difficult Patients<br>• Negative Work Environment<br>• Lack of Clinical Autonomy	"Write about an experience or a physician-patient relationship you have had as a physician that you regret."
6	*When Breath Becomes Air* by Paul Kalanithi, MD	Memoir	256 Pages	• Lack of or Poor Sleep<br>• Mental Illness and Burnout<br>• Staying Physically Healthy<br>• Loneliness and Social Isolation<br>• Moral Distress and Empathy Fatigue<br>• Sick/Dying and Difficult Patients<br>• Work-Life Balance<br>• Unusual, Variable, and Uncertain Work Schedule<br>• High Work Load and Expectations<br>• Career Planning and (Lack of) Professional Support	"What guides you in life besides your duty to your patients? What gives your life meaning outside of medicine?"

For Peer Review Only

Table S2: Pre-Intervention Survey Questions (T_0)

Question	Answer Choices/Format
Survey ID	Free text
Demographic Data	
Gender:	○ Male<br>○ Female<br>○ Other
Your Age (years):	Free text
Race/Ethnicity (select all that apply):	○ White<br>○ Black or African American<br>○ Asian<br>○ Native Hawaiian or other Pacific Islander<br>○ American Indian or Alaska Native<br>○ Hispanic/Latino, or of Spanish Origin<br>○ Multiple Race/Ethnicity not listed above<br>○ Unknown Race/Ethnicity<br>○ Prefer not to answer
Are you an international medical graduate (US citizens and non-US citizens who graduated from any medical school outside of the US and Canada)?	○ Yes<br>○ No<br>○ Prefer not to answer
What is your marital status?	○ Single<br>○ Married<br>○ Non-married Partner<br>○ Divorced
Do you have any children?	○ Yes<br>○ No<br>○ Expecting
How many hours per week did you average at work over the last month?	Free text (limited to integers)
Resident type:	○ Categorical Pediatrics<br>○ Medicine Pediatrics<br>○ Combined Program (i.e. Peds Neuro; Peds Genetics; Peds PMR, Peds Psychiatry; Peds Emergency)
When did you have your last full weekend (Friday evening through Sunday night) off?	○ The previous weekend<br>○ 2 weekends ago<br>○ 3 weekends ago<br>○ 4 or more weekends ago
When was your last vacation (>5 days)?	○ In the past month<br>○ 1-3 months ago<br>○ >3 months ago
What kind of rotation are you on now?	○ Advocacy Elective<br>○ Away/Rural/Public Health Elective<br>○ Elective<br>○ ER<br>○ Global Health Elective<br>○ ICU<br>○ Inpatient<br>○ Jeopardy/Float<br>○ Newborn<br>○ Nightshift Team or Night Float<br>○ Primary Care

For Peer Review Only

Do you or will you participate in one of the following formal Residency Pathways/Tracks/Curricula? (select all that apply)	○ Global Health<br>○ Community Pediatrics and Advocacy<br>○ Integrated Research Pathway<br>○ Primary Care<br>○ Osteopathic Pediatrics<br>○ Other<br>○ None of the above
Have you cared for any children who died during this or your last rotation?	○ Yes<br>○ No
Maslach Burnout Inventory – Two Item[4]	
I feel burned out from my work.	○ Every day<br>○ A few times a week<br>○ Once a week<br>○ A few times a month<br>○ Once a month or less<br>○ A few times a year<br>○ Never
I have become more callous toward people since I took this job.	○ Every day<br>○ A few times a week<br>○ Once a week<br>○ A few times a month<br>○ Once a month or less<br>○ A few times a year<br>○ Never
Perceived Stress Scale[5,6]	
In the last month, how often have you been upset because of something that happened unexpectedly?	○ Never<br>○ Rarely<br>○ Sometimes<br>○ Often<br>○ Always
In the last month, how often have you felt that you were unable to control the important things in your life?	○ Never<br>○ Rarely<br>○ Sometimes<br>○ Often<br>○ Always
In the last month, how often have you felt nervous and “stressed”?	○ Never<br>○ Rarely<br>○ Sometimes<br>○ Often<br>○ Always
In the last month, how often have you felt confident about your ability to handle your personal problems?	○ Never<br>○ Rarely<br>○ Sometimes<br>○ Often<br>○ Always
In the last month, how often have you felt that things were going your way?	○ Never<br>○ Rarely<br>○ Sometimes<br>○ Often<br>○ Always
In the last month, how often have you found that you could not cope with all the things that you had to do?	○ Never<br>○ Rarely<br>○ Sometimes<br>○ Often

For Peer Review Only

	○ Always
In the last month, how often have you been able to control irritations in your life?	○ Never<br>○ Rarely<br>○ Sometimes<br>○ Often<br>○ Always
In the last month, how often have you felt that you were on top of things?	○ Never<br>○ Rarely<br>○ Sometimes<br>○ Often<br>○ Always
In the last month, how often have you been angered because of things that were outside of your control?	○ Never<br>○ Rarely<br>○ Sometimes<br>○ Often<br>○ Always
In the last month, how often have you felt difficulties were piling up so high that you could not overcome them?	○ Never<br>○ Rarely<br>○ Sometimes<br>○ Often<br>○ Always
Cognitive and Affective Mindfulness Scale, R[7]	
It is easy for me to concentrate on what I am doing.	○ Rarely/Not at all<br>○ Sometimes<br>○ Often<br>○ Almost Always
I can tolerate emotional pain.	○ Rarely/Not at all<br>○ Sometimes<br>○ Often<br>○ Almost Always
I can accept things I cannot change.	○ Rarely/Not at all<br>○ Sometimes<br>○ Often<br>○ Almost Always
I can usually describe how I feel at the moment in considerable detail.	○ Rarely/Not at all<br>○ Sometimes<br>○ Often<br>○ Almost Always
I am easily distracted.	○ Rarely/Not at all<br>○ Sometimes<br>○ Often<br>○ Almost Always
It's easy for me to keep track of my thoughts and feelings.	○ Rarely/Not at all<br>○ Sometimes<br>○ Often<br>○ Almost Always
I try to notice my thoughts without judging them.	○ Rarely/Not at all<br>○ Sometimes<br>○ Often<br>○ Almost Always
I am able to accept the thoughts and feelings I have.	○ Rarely/Not at all<br>○ Sometimes<br>○ Often<br>○ Almost Always

For Peer Review Only

I am able to focus on the present moment.	○ Rarely/Not at all<br>○ Sometimes<br>○ Often<br>○ Almost Always
I am able to pay close attention to one thing for a long period of time.	○ Rarely/Not at all<br>○ Sometimes<br>○ Often<br>○ Almost Always
Neff's Self Compassion Scale – Short Form[8]	
When I fail at something important to me, I become consumed by feelings of inadequacy.	○ Never<br>○ Rarely<br>○ Sometimes<br>○ Often<br>○ Always
I try to be understanding and patient towards those aspects of my personality I don't like.	○ Never<br>○ Rarely<br>○ Sometimes<br>○ Often<br>○ Always
When something painful happens, I try to take a balanced view of the situation.	○ Never<br>○ Rarely<br>○ Sometimes<br>○ Often<br>○ Always
When I'm feeling down, I tend to feel like most other people are probably happier than I am.	○ Never<br>○ Rarely<br>○ Sometimes<br>○ Often<br>○ Always
I try to see my failings as part of the human condition.	○ Never<br>○ Rarely<br>○ Sometimes<br>○ Often<br>○ Always
When I'm going through a very hard time, I give myself the caring and tenderness I need.	○ Never<br>○ Rarely<br>○ Sometimes<br>○ Often<br>○ Always
When something upsets me, I try to keep my emotions in balance.	○ Never<br>○ Rarely<br>○ Sometimes<br>○ Often<br>○ Always
When I fail at something that's important to me, I tend to feel alone in my failure.	○ Never<br>○ Rarely<br>○ Sometimes<br>○ Often<br>○ Always
When I'm feeling down, I tend to obsess and fixate on everything that's wrong.	○ Never<br>○ Rarely<br>○ Sometimes<br>○ Often<br>○ Always

For Peer Review Only

When I feel inadequate in some way, I try to remind myself that feelings of inadequacy are shared by most people.	○ Never<br>○ Rarely<br>○ Sometimes<br>○ Often<br>○ Always
I'm disapproving and judgmental about my own flaws and inadequacies.	○ Never<br>○ Rarely<br>○ Sometimes<br>○ Often<br>○ Always
I'm intolerant and impatient towards those aspects of my personality I don't like.	○ Never<br>○ Rarely<br>○ Sometimes<br>○ Often<br>○ Always
Brief Resilience Scale[9]	
I tend to bounce back quickly after hard times.	○ Strongly Disagree<br>○ Disagree<br>○ Neutral<br>○ Agree<br>○ Strongly Agree
I have a hard time making it through stressful events.	○ Strongly Disagree<br>○ Disagree<br>○ Neutral<br>○ Agree<br>○ Strongly Agree
It does not take me long to recover from a stressful event.	○ Strongly Disagree<br>○ Disagree<br>○ Neutral<br>○ Agree<br>○ Strongly Agree
It is hard for me to snap back when something bad happens.	○ Strongly Disagree<br>○ Disagree<br>○ Neutral<br>○ Agree<br>○ Strongly Agree
I usually come through difficult times with little trouble.	○ Strongly Disagree<br>○ Disagree<br>○ Neutral<br>○ Agree<br>○ Strongly Agree
I tend to take a long time to get over setbacks in my life.	○ Strongly Disagree<br>○ Disagree<br>○ Neutral<br>○ Agree<br>○ Strongly Agree
Davis Empathic Concern Scale from the Interpersonal Reactivity Index[10,11]	
When I see people being taken advantage of, I feel kind of protective towards them.	○ Does not describe me (1)<br>○ 2<br>○ 3<br>○ 4<br>○ Describes me well (5)
When I see people being treated unfairly, I sometimes don't feel very much pity for them.	○ Does not describe me (1)<br>○ 2<br>○ 3

For Peer Review Only

	○ 4<br>○ Describes me well (5)
I often have tender, concerned feelings for people less fortunate than me.	○ Does not describe me (1)<br>○ 2<br>○ 3<br>○ 4<br>○ Describes me well (5)
I would describe myself as a pretty soft-hearted person.	○ Does not describe me (1)<br>○ 2<br>○ 3<br>○ 4<br>○ Describes me well (5)
Sometimes I don't feel very sorry for other people when they are having problems.	○ Does not describe me (1)<br>○ 2<br>○ 3<br>○ 4<br>○ Describes me well (5)
Other people's misfortunes do not usually disturb me a great deal.	○ Does not describe me (1)<br>○ 2<br>○ 3<br>○ 4<br>○ Describes me well (5)
I am often quite touched by things I see happen.	○ Does not describe me (1)<br>○ 2<br>○ 3<br>○ 4<br>○ Describes me well (5)
Narrative Medicine Question	
What is your previous experience with narrative medicine?	○ None<br>○ I have participated in a few workshops<br>○ It is a regular practice for me

For Peer Review Only

Table S3: Immediate Post-Intervention Survey Questions (T_1)

Question	Answer Choices/Format
Survey ID	Free text
Demographic Data	
Gender:	○ Male<br>○ Female<br>○ Other
Your Age (years):	Free text
Race/Ethnicity (select all that apply):	○ White<br>○ Black or African American<br>○ Asian<br>○ Native Hawaiian or other Pacific Islander<br>○ American Indian or Alaska Native<br>○ Hispanic/Latino, or of Spanish Origin<br>○ Multiple Race/Ethnicity not listed above<br>○ Unknown Race/Ethnicity<br>○ Prefer not to answer
Are you an international medical graduate (US citizens and non-US citizens who graduated from any medical school outside of the US and Canada)?	○ Yes<br>○ No<br>○ Prefer not to answer
What is your marital status?	○ Single<br>○ Married<br>○ Non-married Partner<br>○ Divorced
Do you have any children?	○ Yes<br>○ No<br>○ Expecting
How many hours per week did you average at work over the last month?	Free text (limited to integers)
Resident type:	○ Categorical Pediatrics<br>○ Medicine Pediatrics<br>○ Combined Program (i.e. Peds Neuro; Peds Genetics; Peds PMR, Peds Psychiatry; Peds Emergency)
When did you have your last full weekend (Friday evening through Sunday night) off?	○ The previous weekend<br>○ 2 weekends ago<br>○ 3 weekends ago<br>○ 4 or more weekends ago
When was your last vacation (>5 days)?	○ In the past month<br>○ 1-3 months ago<br>○ >3 months ago
What kind of rotation are you on now?	○ Advocacy Elective<br>○ Away/Rural/Public Health Elective<br>○ Elective<br>○ ER<br>○ Global Health Elective<br>○ ICU<br>○ Inpatient<br>○ Jeopardy/Float<br>○ Newborn<br>○ Nightshift Team or Night Float<br>○ Primary Care

For Peer Review Only

Do you or will you participate in one of the following formal Residency Pathways/Tracks/Curricula? (select all that apply)	○ Global Health<br>○ Community Pediatrics and Advocacy<br>○ Integrated Research Pathway<br>○ Primary Care<br>○ Osteopathic Pediatrics<br>○ Other<br>○ None of the above
Have you cared for any children who died during this or your last rotation?	○ Yes<br>○ No
Maslach Burnout Inventory – Two Item[4]	
I feel burned out from my work.	○ Every day<br>○ A few times a week<br>○ Once a week<br>○ A few times a month<br>○ Once a month or less<br>○ A few times a year<br>○ Never
I have become more callous toward people since I took this job.	○ Every day<br>○ A few times a week<br>○ Once a week<br>○ A few times a month<br>○ Once a month or less<br>○ A few times a year<br>○ Never
Perceived Stress Scale[5,6]	
In the last month, how often have you been upset because of something that happened unexpectedly?	○ Never<br>○ Rarely<br>○ Sometimes<br>○ Often<br>○ Always
In the last month, how often have you felt that you were unable to control the important things in your life?	○ Never<br>○ Rarely<br>○ Sometimes<br>○ Often<br>○ Always
In the last month, how often have you felt nervous and "stressed"?	○ Never<br>○ Rarely<br>○ Sometimes<br>○ Often<br>○ Always
In the last month, how often have you felt confident about your ability to handle your personal problems?	○ Never<br>○ Rarely<br>○ Sometimes<br>○ Often<br>○ Always
In the last month, how often have you felt that things were going your way?	○ Never<br>○ Rarely<br>○ Sometimes<br>○ Often<br>○ Always
In the last month, how often have you found that you could not cope with all the things that you had to do?	○ Never<br>○ Rarely<br>○ Sometimes<br>○ Often

For Peer Review Only

	○ Always
In the last month, how often have you been able to control irritations in your life?	○ Never<br>○ Rarely<br>○ Sometimes<br>○ Often<br>○ Always
In the last month, how often have you felt that you were on top of things?	○ Never<br>○ Rarely<br>○ Sometimes<br>○ Often<br>○ Always
In the last month, how often have you been angered because of things that were outside of your control?	○ Never<br>○ Rarely<br>○ Sometimes<br>○ Often<br>○ Always
In the last month, how often have you felt difficulties were piling up so high that you could not overcome them?	○ Never<br>○ Rarely<br>○ Sometimes<br>○ Often<br>○ Always
Cognitive and Affective Mindfulness Scale, R[7]	
It is easy for me to concentrate on what I am doing.	○ Rarely/Not at all<br>○ Sometimes<br>○ Often<br>○ Almost Always
I can tolerate emotional pain.	○ Rarely/Not at all<br>○ Sometimes<br>○ Often<br>○ Almost Always
I can accept things I cannot change.	○ Rarely/Not at all<br>○ Sometimes<br>○ Often<br>○ Almost Always
I can usually describe how I feel at the moment in considerable detail.	○ Rarely/Not at all<br>○ Sometimes<br>○ Often<br>○ Almost Always
I am easily distracted.	○ Rarely/Not at all<br>○ Sometimes<br>○ Often<br>○ Almost Always
It's easy for me to keep track of my thoughts and feelings.	○ Rarely/Not at all<br>○ Sometimes<br>○ Often<br>○ Almost Always
I try to notice my thoughts without judging them.	○ Rarely/Not at all<br>○ Sometimes<br>○ Often<br>○ Almost Always
I am able to accept the thoughts and feelings I have.	○ Rarely/Not at all<br>○ Sometimes<br>○ Often<br>○ Almost Always

For Peer Review Only

I am able to focus on the present moment.	○ Rarely/Not at all<br>○ Sometimes<br>○ Often<br>○ Almost Always
I am able to pay close attention to one thing for a long period of time.	○ Rarely/Not at all<br>○ Sometimes<br>○ Often<br>○ Almost Always
Neff's Self Compassion Scale – Short Form[8]	
When I fail at something important to me, I become consumed by feelings of inadequacy.	○ Never<br>○ Rarely<br>○ Sometimes<br>○ Often<br>○ Always
I try to be understanding and patient towards those aspects of my personality I don't like.	○ Never<br>○ Rarely<br>○ Sometimes<br>○ Often<br>○ Always
When something painful happens, I try to take a balanced view of the situation.	○ Never<br>○ Rarely<br>○ Sometimes<br>○ Often<br>○ Always
When I'm feeling down, I tend to feel like most other people are probably happier than I am.	○ Never<br>○ Rarely<br>○ Sometimes<br>○ Often<br>○ Always
I try to see my failings as part of the human condition.	○ Never<br>○ Rarely<br>○ Sometimes<br>○ Often<br>○ Always
When I'm going through a very hard time, I give myself the caring and tenderness I need.	○ Never<br>○ Rarely<br>○ Sometimes<br>○ Often<br>○ Always
When something upsets me, I try to keep my emotions in balance.	○ Never<br>○ Rarely<br>○ Sometimes<br>○ Often<br>○ Always
When I fail at something that's important to me, I tend to feel alone in my failure.	○ Never<br>○ Rarely<br>○ Sometimes<br>○ Often<br>○ Always
When I'm feeling down, I tend to obsess and fixate on everything that's wrong.	○ Never<br>○ Rarely<br>○ Sometimes<br>○ Often<br>○ Always

For Peer Review Only

When I feel inadequate in some way, I try to remind myself that feelings of inadequacy are shared by most people.	○ Never<br>○ Rarely<br>○ Sometimes<br>○ Often<br>○ Always
I'm disapproving and judgmental about my own flaws and inadequacies.	○ Never<br>○ Rarely<br>○ Sometimes<br>○ Often<br>○ Always
I'm intolerant and impatient towards those aspects of my personality I don't like.	○ Never<br>○ Rarely<br>○ Sometimes<br>○ Often<br>○ Always
Brief Resilience Scale[9]	
I tend to bounce back quickly after hard times.	○ Strongly Disagree<br>○ Disagree<br>○ Neutral<br>○ Agree<br>○ Strongly Agree
I have a hard time making it through stressful events.	○ Strongly Disagree<br>○ Disagree<br>○ Neutral<br>○ Agree<br>○ Strongly Agree
It does not take me long to recover from a stressful event.	○ Strongly Disagree<br>○ Disagree<br>○ Neutral<br>○ Agree<br>○ Strongly Agree
It is hard for me to snap back when something bad happens.	○ Strongly Disagree<br>○ Disagree<br>○ Neutral<br>○ Agree<br>○ Strongly Agree
I usually come through difficult times with little trouble.	○ Strongly Disagree<br>○ Disagree<br>○ Neutral<br>○ Agree<br>○ Strongly Agree
I tend to take a long time to get over setbacks in my life.	○ Strongly Disagree<br>○ Disagree<br>○ Neutral<br>○ Agree<br>○ Strongly Agree
Davis Empathic Concern Scale from the Interpersonal Reactivity Index[10,11]	
When I see people being taken advantage of, I feel kind of protective towards them.	○ Does not describe me (1)<br>○ 2<br>○ 3<br>○ 4<br>○ Describes me well (5)
When I see people being treated unfairly, I sometimes don't feel very much pity for them.	○ Does not describe me (1)<br>○ 2<br>○ 3

For Peer Review Only

	○ 4<br>○ Describes me well (5)
I often have tender, concerned feelings for people less fortunate than me.	○ Does not describe me (1)<br>○ 2<br>○ 3<br>○ 4<br>○ Describes me well (5)
I would describe myself as a pretty soft-hearted person.	○ Does not describe me (1)<br>○ 2<br>○ 3<br>○ 4<br>○ Describes me well (5)
Sometimes I don't feel very sorry for other people when they are having problems.	○ Does not describe me (1)<br>○ 2<br>○ 3<br>○ 4<br>○ Describes me well (5)
Other people's misfortunes do not usually disturb me a great deal.	○ Does not describe me (1)<br>○ 2<br>○ 3<br>○ 4<br>○ Describes me well (5)
I am often quite touched by things I see happen.	○ Does not describe me (1)<br>○ 2<br>○ 3<br>○ 4<br>○ Describes me well (5)
Narrative Medicine Intervention Questions	
What are your overall thoughts about this intervention?	Free text
How do you feel that you benefited from participation?	Free text
How do you feel that you can use narrative medicine personally and/or professionally for your wellness at work and at home?	Free text
How can the intervention be improved?	Free text
Do you have any other comments or feedback?	Free text

For Peer Review Only

Table S4: Six Months Post-Intervention Survey Questions (T_2)

Question	Answer Choices/Format
Survey ID	Free text
Demographic Data	
Gender:	○ Male<br>○ Female<br>○ Other
Your Age (years):	○ Free text
Race/Ethnicity (select all that apply):	○ White<br>○ Black or African American<br>○ Asian<br>○ Native Hawaiian or other Pacific Islander<br>○ American Indian or Alaska Native<br>○ Hispanic/Latino, or of Spanish Origin<br>○ Multiple Race/Ethnicity not listed above<br>○ Unknown Race/Ethnicity<br>○ Prefer not to answer
Are you an international medical graduate (US citizens and non-US citizens who graduated from any medical school outside of the US and Canada)?	○ Yes<br>○ No<br>○ Prefer not to answer
What is your marital status?	○ Single<br>○ Married<br>○ Non-married Partner<br>○ Divorced
Do you have any children?	○ Yes<br>○ No<br>○ Expecting
How many hours per week did you average at work over the last month?	○ Free text (limited to integers)
Resident type:	○ Categorical Pediatrics<br>○ Medicine Pediatrics<br>○ Combined Program (i.e. Peds Neuro; Peds Genetics; Peds PMR, Peds Psychiatry; Peds Emergency)
When did you have your last full weekend (Friday evening through Sunday night) off?	○ The previous weekend<br>○ 2 weekends ago<br>○ 3 weekends ago<br>○ 4 or more weekends ago
When was your last vacation (>5 days)?	○ In the past month<br>○ 1-3 months ago<br>○ >3 months ago
What kind of rotation are you on now?	○ Advocacy Elective<br>○ Away/Rural/Public Health Elective<br>○ Elective<br>○ ER<br>○ Global Health Elective<br>○ ICU<br>○ Inpatient<br>○ Jeopardy/Float<br>○ Newborn<br>○ Nightshift Team or Night Float<br>○ Primary Care

For Peer Review Only

Do you or will you participate in one of the following formal Residency Pathways/Tracks/Curricula? (select all that apply)	○ Global Health<br>○ Community Pediatrics and Advocacy<br>○ Integrated Research Pathway<br>○ Primary Care<br>○ Osteopathic Pediatrics<br>○ Other<br>○ None of the above
Have you cared for any children who died during this or your last rotation?	○ Yes<br>○ No
Maslach Burnout Inventory – Two Item[4]	
I feel burned out from my work.	○ Every day<br>○ A few times a week<br>○ Once a week<br>○ A few times a month<br>○ Once a month or less<br>○ A few times a year<br>○ Never
I have become more callous toward people since I took this job.	○ Every day<br>○ A few times a week<br>○ Once a week<br>○ A few times a month<br>○ Once a month or less<br>○ A few times a year<br>○ Never
Perceived Stress Scale[5,6]	
In the last month, how often have you been upset because of something that happened unexpectedly?	○ Never<br>○ Rarely<br>○ Sometimes<br>○ Often<br>○ Always
In the last month, how often have you felt that you were unable to control the important things in your life?	○ Never<br>○ Rarely<br>○ Sometimes<br>○ Often<br>○ Always
In the last month, how often have you felt nervous and "stressed"?	○ Never<br>○ Rarely<br>○ Sometimes<br>○ Often<br>○ Always
In the last month, how often have you felt confident about your ability to handle your personal problems?	○ Never<br>○ Rarely<br>○ Sometimes<br>○ Often<br>○ Always
In the last month, how often have you felt that things were going your way?	○ Never<br>○ Rarely<br>○ Sometimes<br>○ Often<br>○ Always
In the last month, how often have you found that you could not cope with all the things that you had to do?	○ Never<br>○ Rarely<br>○ Sometimes<br>○ Often

For Peer Review Only

	○ Always
In the last month, how often have you been able to control irritations in your life?	○ Never<br>○ Rarely<br>○ Sometimes<br>○ Often<br>○ Always
In the last month, how often have you felt that you were on top of things?	○ Never<br>○ Rarely<br>○ Sometimes<br>○ Often<br>○ Always
In the last month, how often have you been angered because of things that were outside of your control?	○ Never<br>○ Rarely<br>○ Sometimes<br>○ Often<br>○ Always
In the last month, how often have you felt difficulties were piling up so high that you could not overcome them?	○ Never<br>○ Rarely<br>○ Sometimes<br>○ Often<br>○ Always
Cognitive and Affective Mindfulness Scale, R[7]	
It is easy for me to concentrate on what I am doing.	○ Rarely/Not at all<br>○ Sometimes<br>○ Often<br>○ Almost Always
I can tolerate emotional pain.	○ Rarely/Not at all<br>○ Sometimes<br>○ Often<br>○ Almost Always
I can accept things I cannot change.	○ Rarely/Not at all<br>○ Sometimes<br>○ Often<br>○ Almost Always
I can usually describe how I feel at the moment in considerable detail.	○ Rarely/Not at all<br>○ Sometimes<br>○ Often<br>○ Almost Always
I am easily distracted.	○ Rarely/Not at all<br>○ Sometimes<br>○ Often<br>○ Almost Always
It's easy for me to keep track of my thoughts and feelings.	○ Rarely/Not at all<br>○ Sometimes<br>○ Often<br>○ Almost Always
I try to notice my thoughts without judging them.	○ Rarely/Not at all<br>○ Sometimes<br>○ Often<br>○ Almost Always
I am able to accept the thoughts and feelings I have.	○ Rarely/Not at all<br>○ Sometimes<br>○ Often<br>○ Almost Always

For Peer Review Only

I am able to focus on the present moment.	○ Rarely/Not at all<br>○ Sometimes<br>○ Often<br>○ Almost Always
I am able to pay close attention to one thing for a long period of time.	○ Rarely/Not at all<br>○ Sometimes<br>○ Often<br>○ Almost Always
Neff's Self Compassion Scale – Short Form[8]	
When I fail at something important to me, I become consumed by feelings of inadequacy.	○ Never<br>○ Rarely<br>○ Sometimes<br>○ Often<br>○ Always
I try to be understanding and patient towards those aspects of my personality I don't like.	○ Never<br>○ Rarely<br>○ Sometimes<br>○ Often<br>○ Always
When something painful happens, I try to take a balanced view of the situation.	○ Never<br>○ Rarely<br>○ Sometimes<br>○ Often<br>○ Always
When I'm feeling down, I tend to feel like most other people are probably happier than I am.	○ Never<br>○ Rarely<br>○ Sometimes<br>○ Often<br>○ Always
I try to see my failings as part of the human condition.	○ Never<br>○ Rarely<br>○ Sometimes<br>○ Often<br>○ Always
When I'm going through a very hard time, I give myself the caring and tenderness I need.	○ Never<br>○ Rarely<br>○ Sometimes<br>○ Often<br>○ Always
When something upsets me, I try to keep my emotions in balance.	○ Never<br>○ Rarely<br>○ Sometimes<br>○ Often<br>○ Always
When I fail at something that's important to me, I tend to feel alone in my failure.	○ Never<br>○ Rarely<br>○ Sometimes<br>○ Often<br>○ Always
When I'm feeling down, I tend to obsess and fixate on everything that's wrong.	○ Never<br>○ Rarely<br>○ Sometimes<br>○ Often<br>○ Always

For Peer Review Only

When I feel inadequate in some way, I try to remind myself that feelings of inadequacy are shared by most people.	○ Never<br>○ Rarely<br>○ Sometimes<br>○ Often<br>○ Always
I'm disapproving and judgmental about my own flaws and inadequacies.	○ Never<br>○ Rarely<br>○ Sometimes<br>○ Often<br>○ Always
I'm intolerant and impatient towards those aspects of my personality I don't like.	○ Never<br>○ Rarely<br>○ Sometimes<br>○ Often<br>○ Always
Brief Resilience Scale[9]	
I tend to bounce back quickly after hard times.	○ Strongly Disagree<br>○ Disagree<br>○ Neutral<br>○ Agree<br>○ Strongly Agree
I have a hard time making it through stressful events.	○ Strongly Disagree<br>○ Disagree<br>○ Neutral<br>○ Agree<br>○ Strongly Agree
It does not take me long to recover from a stressful event.	○ Strongly Disagree<br>○ Disagree<br>○ Neutral<br>○ Agree<br>○ Strongly Agree
It is hard for me to snap back when something bad happens.	○ Strongly Disagree<br>○ Disagree<br>○ Neutral<br>○ Agree<br>○ Strongly Agree
I usually come through difficult times with little trouble.	○ Strongly Disagree<br>○ Disagree<br>○ Neutral<br>○ Agree<br>○ Strongly Agree
I tend to take a long time to get over setbacks in my life.	○ Strongly Disagree<br>○ Disagree<br>○ Neutral<br>○ Agree<br>○ Strongly Agree
Davis Empathic Concern Scale from the Interpersonal Reactivity Index[10,11]	
When I see people being taken advantage of, I feel kind of protective towards them.	○ Does not describe me (1)<br>○ 2<br>○ 3<br>○ 4<br>○ Describes me well (5)
When I see people being treated unfairly, I sometimes don't feel very much pity for them.	○ Does not describe me (1)<br>○ 2<br>○ 3

For Peer Review Only

	○ 4<br>○ Describes me well (5)
I often have tender, concerned feelings for people less fortunate than me.	○ Does not describe me (1)<br>○ 2<br>○ 3<br>○ 4<br>○ Describes me well (5)
I would describe myself as a pretty soft-hearted person.	○ Does not describe me (1)<br>○ 2<br>○ 3<br>○ 4<br>○ Describes me well (5)
Sometimes I don't feel very sorry for other people when they are having problems.	○ Does not describe me (1)<br>○ 2<br>○ 3<br>○ 4<br>○ Describes me well (5)
Other people's misfortunes do not usually disturb me a great deal.	○ Does not describe me (1)<br>○ 2<br>○ 3<br>○ 4<br>○ Describes me well (5)
I am often quite touched by things I see happen.	○ Does not describe me (1)<br>○ 2<br>○ 3<br>○ 4<br>○ Describes me well (5)
Narrative Medicine Intervention Questions	
6 months later, what are your overall thoughts about this intervention?	Free text
6 months later, how do you feel that you benefited from participation?	Free text
6 months later, how do you feel that you can use narrative medicine personally and/or professionally for your wellness at work and at home?	Free text
Do you have any other comments or feedback?	Free text

For Peer Review Only

Table S5: Baseline demographic data of participants in the intervention group who took the initial survey in April versus December 2020

Category	Group	April 2020 n (%)	December 2020 n (%)	p-value
**Gender**	Male	2 (20)	3 (27)	0.70
	Female	8 (80)	8 (73)	
	Total	10	11	
**Age**	<30	10 (100)	8 (73)	0.07
	30+	0 (0)	3 (27)	
	Total	10	11	
**International Medical Education**	Yes	0 (0)	1 (9)	0.31
	No	11 (100)	10 (91)	
	Total	11	11	
**Marital Status**	Single/divorced	4 (36)	6 (55)	0.39
	Married/partnered	7 (64)	5 (45)	
	Total	11	11	
**Children**	Yes	0 (0)	2 (18)	0.16
	No	10 (100)	9 (82)	
	Total	10	11	
**Categorical**	Yes	10 (91)	6 (55)	0.08
	No	1 (9)	5 (45)	
	Total	11	11	
**Last Weekend Off**	Previous weekend	5 (45)	4 (36)	0.86
	2 weekends ago	4 (36)	4 (36)	
	3+ weekends ago	2 (18)	3 (27)	
	Total	11	11	
**Last Vacation**	In the past month	5 (45)	4 (36)	0.63
	1-3 months ago	2 (18)	4 (36)	
	>3 months ago	4 (36)	3 (27)	
	Total	11	11	
**Last Rotation**	Inpatient/Newborn/Nightshift	6 (60)	6 (55)	0.91
	ER/ICU	2 (20)	2 (18)	
	Primary Care/Elective	2 (20)	3 (27)	
	Total	10	11	
**Patient Death**	Yes	0 (0)	2 (18)	0.14
	No	11 (100)	9 (82)	
	Total	11	11	

Note: n may not sum to total sample size because of missing data. Percentages may not sum to 100% due to rounding.

For Peer Review Only
